# Treatment of a Multitraumatized Tortured Refugee Needing an Interpreter with Exposure Therapy

**DOI:** 10.1155/2013/197323

**Published:** 2013-02-06

**Authors:** Bo Søndergaard Jensen

**Affiliations:** Clinic for PTSD and Transcultural Psychiatry, Aarhus University Hospital, Skejbygaardsvej 13-17, 8240 Rissikov, Denmark

## Abstract

This paper described the application and feasibility of exposure therapy treatment (ET) for posttraumatic stress disorder (PTSD) in a multitraumatized tortured refugee with chronic PTSD and depression, in need of an interpreter. The patient received 26 one-hour sessions of ET involving exposure to his trauma-related memories. Symptoms of PTSD, depression, and anxiety were assessed at pre- and posttreatment and 3-, 6-, and 12-month followup with the Harvard Trauma Questionnaire (HTQ-R), PTSD Symptom Scale-Self Report (PSS-SR), Major depression inventory (MDI), and Beck Anxiety Inventory (BAI). Treatment led to a significant improvement across all measures of posttraumatic stress disorder, anxiety, and depression, and the improvement was maintained at the 12-month follow-up. The results from this case study provide further preliminary evidence that ET may be effective in treating multi-traumatized torture survivors who are refugees and in need of an interpreter, despite the additional stressors and symptoms complexity experienced by tortured refugees.

## 1. Introduction

PTSD is a highly prevalent, often chronic, and invalidating psychiatric disorder that can develop following exposure to a traumatic event. It is a condition resulting from exposure to a life threatening event that is processed in such a way that the experience produces a sense of a current threat and two broad sets of negative cognitions, “The world is entirely dangerous” and “I am completely incompetent to cope with it” [1, 2]. This leads to the experiencing of symptoms that fall into three clusters and define PTSD according to the diagnostic systems in DSM-IV-TR and ICD-10: intrusive symptoms, avoidance symptoms, and symptoms of hyperarousal [3, 4].

 Among refugees it is estimated that approximately 3% to 86% of the population suffers from PTSD, depending upon the type of sample, the type of traumatic events, and the refugee groups that were studied [5]. In 2001, The Danish Ministry of Health estimated that at least 50,000 refugees in Denmark suffered from PTSD and/or depression and that 5 to 44% of them are estimated to have a history of torture [6]. In 2008, the Amnesty International Danish Medical Group published a report based on medical examinations of 142 newly arrived asylum seekers in Denmark. Of the newly arrived asylum seekers, 45% said that they had been tortured. The psychological symptoms were approximately three times as frequent among torture survivors as among nontortured asylum seekers. Among torture survivors 63% met the criteria for PTSD, with the study suggesting that torture survivors with severe physical and psychological symptoms represent a significant proportion of newly arrived asylum seekers in Denmark [7].

 In particular, the experience of torture has been shown to have a severe impact on the survivor's mental health, especially in terms of the diagnoses of PTSD and major depression [8]. In their comparison of 160 refugees from six different nations who had been tortured in their homeland, Moisander and Edston [9] found that PTSD ranged from 69% to 92% across all patient groups. According to a report from Amnesty International, 111 countries conducted torture or mistreatment, 55 nations conducted unfair trials, and 96 countries restricted their citizens' freedom of expression, while 48 countries had prisoners of conscience, sitting behind bars [10]. Because of this widespread occurrence of torture, it is important to find and use effective and evidence-based therapies for PTSD among refugees who have been subjected to torture.

 Several empirical reviews have concluded that exposure therapy is a particularly efficacious treatment for PTSD and exposure therapy has generated more empirical evidence in support of its use than any other treatment for PTSD [11–13]. Despite the large body of empirical evidence documenting the effectiveness of exposure therapy compared with other approaches in treating PTSD, individuals with PTSD do not typically receive this treatment. Becker et al. [14] estimated that only 17% of practitioners use exposure-based therapy in the treatment of PTSD, and also concluded that clinicians appear to perceive a significant number of barriers to implementing exposure. One of the main barriers is the persistent concerns that imaginal exposure may exacerbate fear and anxiety, which can lead to a retraumatization of the patients [15]. The concerns about this is receiving support from the interpretation of the results from the case reports and studies by Pitman et al. [16] and Tarrier et al. [17].

Foa et al. [18] conducted a study specifically investigating whether imaginal exposure exacerbate PTSD symptoms. They found that only a minority of the participants, 76 women with chronic PTSD, exhibited reliable symptom exacerbation and individuals who reported symptom exacerbation benefited comparably from treatment. Furthermore, symptom exacerbation was unrelated to dropout. The conclusion in this study was that although a minority of individuals experienced a temporary symptom exacerbation, this exacerbation was unrelated to outcome.

In their review of cognitive behavioural intervention for PTSD, Rothbaum et al. [19] noted that some findings suggest that not everyone can participate in exposure therapy. This group may include trauma survivors who are unwilling to confront trauma reminders or memories or to tolerate temporarily increased levels of anxiety and PTSD symptoms. Rothbaum et al. [19] concluded that even with these limitations, exposure therapy has revived the strongest amount of evidence in support of its efficacy in reducing PTSD and should be considered a first-line intervention. This recommendation is in accordance with the more recent meta-analysis from The Cochrane Database of Systematic Reviews [12] and the Institute of Medicine [13], both of which also point out that exposure therapy has the strongest evidence in support of its efficacy in reducing PTSD.

## 2. Case Presentation

### 2.1. Participant

The patient was a 60-year-old male Iraqi refugee in treatment for chronic PTSD and depression in the psychiatric out-patient clinic for PTSD and Transcultural Psychiatry in Aarhus, Denmark.

The patient had arrived in Denmark in 1993 and reported a history of persecution, arrest, detention, imprisonment, and torture in Iraq. He had been imprisoned for several months each time, including once the 1970s, once the 1980s and once in 1990s. Soon after his release from the last imprisonment, he left Iraq with his family. He reported having been subjected to various forms of torture, including severe beatings, flogging, electric shock, blindfolding, deprivation of food, water, and sleep, verbal abuse, mockery/humiliation, being stripped naked, and being deprived of medical care. 

 His reexperiencing symptoms included nightmares and intrusive images of the torture he had experienced under his imprisonment in the 1970s, 1980s, and 1990s. He showed substantial distress when faced with reminders of the torture and the imprisonment (e.g., residential areas, because it reminded him about the secret prison in Baghdad where he was imprisoned as a political prisoner). Moreover his nightmares about being tortured were very vivid, including smell and bodily sensations. After awakening he felt a painful sensation in the area where he had been whipped. The patient described, “Every morning, I feel the same pain as I did back then”. 

He displayed fear of having nightmares and his sleep pattern was severely disrupted. The patient avoided thinking or talking about the event, avoiding people of Arab descent and residential areas because it reminded him of his imprisonment. He reported significant levels of anger outbursts, difficulty in concentrating, and an exaggerated startle response. Pretreatment, the patient fulfilled the criteria for PTSD related to the torture, as well as having a major depressive disorder. In the period just before starting at the clinic, the patient had attempted to reduce his anxiety and irritability by using alcohol, though at the time he started treatment he had stopped this behaviour. The patient was reassured about confidentiality by emphasizing that information would not be made available to a third party without his permission, and that it would only be used for publication in professional journals. The patient gave informed consent for publication.

### 2.2. Treatment

The treatment manual was developed with in the theoretical framework of the emotional processing theory according to Foa and Kozak [20], the cognitive model of posttraumatic stress disorder according to Ehlers and Clark [1], and narrative exposure therapy according to Schauer et al. [21]. Foa and Kozak developed the emotional processing theory as a theoretical framework for the development of PTSD pathology and ways to correct this pathology in treatment. In this theory, it is assumed that the memory of the traumatic incident is stored in the memory as a fear structure designed as a programme to avoid danger in the future [2, 20]. For treatment to be effective, the pathological elements of the fear structure must be corrected, which can only be achieved if the fear structure is activated and if new information that is incompatible with the existing information in the fear structure is introduced [2]. In addition to the emotional processing theory, cognitive models of PTSD have also emphasized the role of fear activation in effective treatment. For example, Ehlers and Clark [1] underscore the need to relive the trauma in recollection so that elaboration and contextualization of the trauma memory can take place and negative assumptions about recalling the trauma can be tested. Schauer et al. [21] emphasize that patients with PTSD often fail to integrate traumatic experiences into the narrative of their lives. Hence the patient cannot report the traumatic experience in a consistent, chronological order and thus has no explicit link between the various events, life experiences, and context within which the events occurred. The treatment conducted in this case study was individual, the sessions lasted for 60 minutes, and were scheduled once a week for a total treatment period of 10 months. In this 10-month period the patient attended 20 sessions and cancelled six sessions due to sickness.

Sessions 1 to 3 were a combination of initial interview and diagnostic assessment. During these sessions the patient identified the events which were most traumatic in his lifetime and identified the trauma that seemed to be causing him the most distress. This traumatic experience was then selected to be the primary focus of treatment. 

 Session 4-5 were psychoeducation about PTSD and the rationale for the treatment. The patient was told that PTSD symptoms are a common response to extreme and harmful experiences and that imprisonment and torture causes not only injuries to the body, but also to the mind and soul. The patient was told that his memory of the traumatic incident was stored in his memory as a programme to help avoid danger in the future, and that this programme was not an integrated part of his autobiographical memory. Additionally the patient was also told that this unintegrated memory produced the symptoms of PTSD. To make this rationale tangible and visual for the patient, the therapist used the following explanation:

*Imagine that our memory is like this bookcase in my office. We have recollections of past experiences, and these recollections are chronologically stored in our autobiographical memory like the books in this bookcase. We have a recollection about our first day at school (the therapist takes a book from the bookshelf).*

*In this recollection the book has stored all the information about what was happening that day, what we experienced and what we felt at that place at that time in our life. Like this book, we can bring forward the recollection about the event and generate images and emotions from that day. As with reading from the book, and we can put it back where it belongs in our autobiographical memory *(the therapist puts back the book on the bookshelf). **

*We can bring forward another recollection from our life for example, our last day at school, and look at it, and we have then the feeling that this recollection is closer to the present.*

*Unprocessed traumatic memories are not properly stored in our autobiographical memory as they are with this book (the therapist takes a book and places it on top of the other books in the bookcase).*

*Your brain is trying to integrate this memory, but every time it appears you try to avoid thinking about it because it gives you the same feeling as when it happened. As a result, you throw the book back on top of the other books in the bookcase, thus preventing yourself from processing the traumatic memory and storing it in its proper place in your autobiographical memory.*

*In this treatment, we will give you the opportunity to look at your life, especially with this book. This will give you an opportunity to view your life, particularly in relation to the traumatic event and its meaning from your perspective today, knowing that you are safe here, rather than from the perspective of the past when it was still terrifying.*

*So the goal of revisiting the past is to enable you to have thoughts and feelings about the past and about the trauma, to talk and think about it without getting so upset or anxious that it disrupts your life. In this way, we will help you to make sense of the terrible experiences and process the trauma.*

The psycho-education about PTSD and the rationale for the treatment were recorded on an mp3 player, and the patient was instructed to listen to the recording at home one to two times every week during the treatment. In Session 6, a Subjective Units of Discomfort scale (SUD) was constructed in order to monitor how much discomfort or anxiety revisiting of the past was causing the patient in the present. In Session 7, the patient started the construction of a detailed chronological narrative of his biography in cooperation with the therapist. The patient's testimony was written down by the therapist, and in the subsequent sessions, the narrative was read aloud to the patient, who was asked to correct it and add any missing details. During the reading of the narrative to the patient, the therapist asked the patient how much distress, upon hearing the narrative, was causing the patient according to the SUD scale. The SUD ratings were recorded by the therapist in the written testimony, in order to help the therapist better monitor the patient's level of discomfort in subsequent sessions. The SUD ratings during the narration and reading of the narrative were also used to ground the patient in the present, thereby preventing avoidance, dissociation, or flashbacks. Before proceeding with the testimony, the patient and therapist discussed whether any new information had come forward and whether there had been any habituation to the patient's discomfort. During the narration, the patient was asked questions about the contents of his fear structure, on the cognitive, emotional, and physiological levels, to reassure that the traumatic fear structure was activated, so that traumatic experience could be emotionally processed and new information incompatible with the existing information in the fear structure could be introduced. The procedure was repeated across Sessions 7 to 20 until a final version of the patient's trauma narrative was reached. The sessions revealed a long history of persecution of the patient, which included many potentially traumatic events. 

 After the final version of the narrative was created in Session 20, the trauma part of the narrative was translated into Arabic and the patient was instructed to read the trauma narrative at home every other day. He was told to read it three times so that the combined reading of the trauma narrative had a time-related length of 45 minutes. During the reading the patient was instructed to record his SUD level in preprinted fields upon the receipt of the Arabic translation. The preprinted fields were placed in the text in such a way that it was estimated that it would take five minutes to read the section before the next SUD level register-field came into the translation. In the translation, three days' worth of preprinted fields, including three fields per day, were given with instructions to record the SUD level each day. The patient's self-reported SUD level can be found in Table 4.

Sessions 21 to 26 were used to discuss the homework exposure and recorded SUD level. How it was for the patient to revisit the traumatic event, whether there was any habituation of the anxiety level during reading, whether there was some new information that should be added to the trauma narrative, and whether there was some new understanding of the traumatic event.

 The patient was at the beginning of treatment medicated with Paroxetin and Mirtazapin. After 2 month the patient discontinued this medication due to side effects. He did subsequently not receive any pharmacological treatment for his PTSD or depression. During treatment the patient also received 6 session of physiotherapy with focus on pain management. 

### 2.3. Assessment

Psychiatric status was assessed using the Mini-International Neuropsychiatric Interview (M.I.N.I.), which is a short structured diagnostic interview developed by psychiatrists and clinicians in the United States and Europe for DSM-IV and ICD-10 psychiatric disorders. It was designed to meet the need for a short but accurately structured psychiatric interview for multicentre clinical trials and epidemiology studies and has an administration time of approximately 15 minutes [22].

For assessor rating, the revised version of the Harvard Trauma Questionnaire (HTQ-R), Section IV, 16 symptoms corresponding to the criteria for PTSD according to the DSM-III, was used as a structured interview for the symptom severity of PTSD [23].

Data on the psychometric properties of the HTQ are reported in Mollica et al. [24]. Data on the psychometric properties of the HTQ-R have not been published, but based on the changes made from the HTQ to the HTQ-R, it can be assumed that they will be highly similar. In the HTQ-R, the 16 PTSD symptoms remain unchanged. The HTQ have a sensitivity of 93% (i.e., 93% of patients with PTSD were correctly classified by the HTQ) and a specificity of 84% (i.e., 84% of patients without PTSD were correctly classified by the HTQ).

For self-report ratings, the following was used: PTSD Symptom Scale-Self Report (PSS-SR), Beck Anxiety Inventory (BAI), and Major Depression Inventory (MDI). The PSS-SR consist of 17 questions that correspond to PTSD the Diagnostic and Statistical Manual of Mental Disorders (3rd ed., rev., DSM-III-R; American Psychiatric Association, 1987). Each symptom is rated on a four-point scale ranging from 0 (not at all) to 3 (very much). PSS-SR has been found to be internally consistent (Cronbach's *α* = .91) and stable over a period of one month (*r* = .74). Subscales assessing re-experiencing, avoidance, and arousal were also internally consistent and stable [25]. The BAI is a 21-item inventory measure for trait anxiety. It has a good internal consistency, an acceptable reliability, and an acceptable convergent and discriminate validity [26–28]. The MDI has been developed to measure DSM-IV and ICD-10 diagnoses of major (moderate to severe) depression by self-reported symptoms. The MDI can be used as both a measuring instrument and a diagnostic instrument with algorithms leading to the DSM-IV or ICD-10 categories of major or moderate to severe depression [29].

All self-report scales were translated into Arabic by a team of interpreters and clinicians, and were back-translated to check its accuracy. The translation team consisted of two male Arab immigrant from Middle Eastern countries, one from Egypt and one from Iraq, who had an average of approximately 25 years of experience providing written and verbal Arabic translations to local health and social services. The translated forms were compared and satisfied both the interpreters and clinicians. The goal of the translation was a loyalty of meaning and an equal familiarity and colloquialness in Danish, English, and Arabic [30]. 

### 2.4. Outcome

Results from pre- to post-treatment and the 3-, 6-, 12-month follow-up on individual measures can be found in Tables 1 and 2. Results of the ongoing monitoring during treatment can be found in Table 3. The HTQ-R score decreased 54% from pre- to post-treatment, from a total score of 3.44 to a total score of 1.56. A score of 1.56 on the HTQ-R is below the threshold, whereas individuals with an HTQ-R score of 2.5 or higher are considered symptomatic for PTSD [23]. During the follow-up period the score of the HTQ-R stayed below the threshold at the 3-, 6-, and 12-month follow-up.

The PSS-SR score decreased 66% from pre- to post-treatment, from a total score of 44 in the extremely severe range of 41 to 51, to a total score of 14 in the subclinical to mild range from 11 to 15. At the 3-month follow-up, there was an increase in the PSS-SR to a total score of 20 in the mild range from 16 to 20. At the 6-month follow-up there was a decrease in the PSS-SR to a total score of 10 in the below threshold range from 0 to10, and to a total score af 4 in the 12-month follow-up.

The BAI score decreased 63% from pre- to post-treatment, from a score of 46, indicating a high anxiety to a score of 17, indicating a very low anxiety. During the follow-up period the score of the BAI stayed under 21, thus indicating a very low anxiety. The MDI score decreased 41% from pre- to post-treatment, from a score of 39 in the range of severe depression of 30 to 50, to a score of 23 in the range of mild depression from 20 to 24. At the 3-month follow-up, there was an increase in the MDI to a score of 25 in the range of moderate depression from 25 to 29. At the 6-month follow-up there was a decrease in the MDI to a score of 19 in the below threshold range from 0 to 19, while at 12-month followup there was a score of 9, indicating no depression.

## 3. Discussion

Some authors have suggested that PTSD symptoms following multiple traumatic events, in particular those originating from organized violence and torture or other forms of prolonged repeated trauma, are different form PTSD symptoms following single events such as motor vehicle accidents, rape, and so forth. As a result of this, these patients should be treated differently and that they would not profit from treatment involving exposure [31–33].

In this case study, it was assumed on the basis of new research literature that ET could be used to reduce/treat PTSD in a severe traumatized refugee. Improvement was achieved in a relatively short period of time (26 sessions lasting 1 hour each). At 3-month follow-up, the patient reported about increase in symptoms. The PSS-SSR score was increased to a total score of 20, indicating mild PTSD, but there were also an increase in the MDI score to 25, thereby indication moderate depression. In spite of this increase of symptoms in terms of the PSS-SR and MDI score, the BAI stayed in the range below 21, indicating a low level of anxiety. The HTQ-R also stayed below the threshold of 2.5, indicating no PTSD. One possible explanation for this rating could be that although the patient experienced a heightening of symptoms severity measured on the PSS-SR and the MDI, this did not lead to a significant increase in the subjectively experienced discomfort and anxiety measured with the HTQ-R and BAI, because the patient had learned to emotionally deal with the trauma and its reminders through the treatment. This could also be part of the explanation for the further reduction in symptoms that appeared at 6- and 12-month follow-up, in which all measures were below the threshold, indicating subclinical or none PTSD or depression. 

This symptom reduction also continued between the 6- and 12-month follow-up, hence indicating a sustainable change in the psychopathology (Tables 1 and 2).

 Another interesting finding is the decrease of the PSS-SR score between Session 20 and Session 24 (Table 3). One possible explanation for this decrease could be that the decline appeared after the patient was instructed to read the translated narrative at home so many times that the combined reading of the trauma narrative had a time-related extent of 45 minutes. In support of this hypothesis is also the fact that there is a coincidence between the decrease in the PSS-SR score and the decrease in the subjectively experienced discomfort measured with SUD (Table 4) during the reading of the trauma narrative. This is in accordance with the research carried out in relation to the treatment of PTSD with Prolonged Exposure Therapy [2], in which a correlation between the prolonged and repeated confrontation/exposure to the trauma memory and the treatment efficacy is present. Furthermore, this could be a possible explanation for the fact that this case study exhibited better treatment outcomes than that found by Halvorsen and Stenmark [34] in their study of 16 torture survivors, who received 10 sessions of narrative exposure therapy (NET). In this study, they found moderate treatment gains in symptoms of chronic PTSD in severely traumatized refugee survivors of torture; between 40% and 65% no longer met the diagnostic criteria for PTSD at follow-up, depending on the score rule used for Clinician-Administered PTSD Scale (CAPS).

The case study showed preliminary evidence that ET can be useful in reducing symptoms of PTSD, depression, and anxiety in a refugee who had experienced multiple traumatic events over long periods of time, including torture. This is consistent with the results of an open clinical trial [35] involving 16 tortured refugees in Sweden, who were successfully treated with cognitive-behavior therapy (CBT), as well as with the findings of Robjant and Fazel [36] that NET can be effective among individuals who have experienced multiple, repeated traumatic events, in reducing the rates and severity of PTSD. It can not be ruled out that a large proportion of the treatment effect can be attributed to nonspecific factors and not to the specific method. Nonspecific factors such as the therapeutic alliance and perceived social support could have influenced the outcome of the psychotherapy more than the specific intervention. However, the finding of a decrease in the PSS-SR, and SUD score between Session 20 and Session 26 (Tables 3 and 4) might indicate that the exposure component is of great importance because this significant reduction in the symptom level first appeared after the patient was instructed to read the translated narrative at home so many times that the combined reading of the trauma narrative had a time-related length of 45 minutes.

Clearly, results from a case study have limited generalization, and there is a need for further research into the use of exposure based treatment methods for PTSD in refugees who have experienced multiple traumatic events over long periods, including torture. The importance of such further research is also emphasized with respect to the previous finding from Carlsson et al. [37] and Birck [38] regarding the results of treatment for traumatized refugees. Carlsson et al. [37] examined the outcome of a multidisciplinary treatment, including psychotherapy, physiotherapy, and social assistance, among 55 torture survivors. The main finding of the study was no improvement in the symptoms of PTSD, depression, anxiety or health-related quality of life after a mean of eight months of treatment. Birck [38] conducted a 2-year follow-up of 30 torture survivors who had received a mean duration of mostly psychodynamic-orientated therapy of 23.5 months. At the 2-year follow-up, there was no significant decrease in symptoms of depression and anxiety, in addition to no decrease of PTSD, except for a significant decrease in the symptoms of intrusion. There is therefore a need to continue to explore new and more effective methods for the treatment of refugees needing an interpreter who have experienced multiple traumatic events over long periods, including torture.

In addition, if further research is conducted in a general psychiatric clinic with outpatient treatment, this can strengthen the general evaluation regarding the utility and capacity of an intervention to both reduce psychopathology under controlled conditions (efficacy) and to be successful when implemented in routine treatment settings (effectiveness). Further systematic study of this field can also be in the interest of refugees, internally displaced persons, and the countries that are faced with the challenge of providing mental health care to these persons. This is indeed a serious challenge for the world community, given that according to recent reports from the United Nations High Commissioner for Refugees, there are approximately 43.7 million forcibly displaced people worldwide. Of these, 27.5 million were internally displeased persons, 15. 4 million were refugees, and 837,500 asylum seekers [39].

## Figures and Tables

**Table 1:** Scores on clinical assessor rating measures at pre- and posttreatment and follow-up.

	Pretreatment	Posttreatment	3 months	6 months	12 months
HTQ-R re-experiencing score	4	1.5	1.5	1.5	1
HTQ-R avoidance and numbing score	3.29	1.29	1.57	1.29	1
HTQ-R alertness	3.2	2	2.4	2	1.6
HTQ-R total score	3.44	1.56	1.81	1.56	1.19

**Table 2:** Scores on clinical self-report measures at pre- and post-treatment and follow-up.

	Pretreatment	Posttreatment	3 months	6 months	12 months
PSS-SR	42	14	20	10	4
BAI	46	17	20	13	5
MDI	39	23	25	19	9

**Table 3:** Results of the ongoing monitoring of treatment efficacy during treatment.

	Session 2	Session 12	Session 16	Session 20	Session 24	Session 26
PSS-SR	42	33	34	34	18	14

**Table:** (a) The recording between Sessions 21 and 22

	Field 1	Field 2	Field 3	Field 4	Field 5
Day 1					
(1) Reading	50	50	70	80	70
(2) Reading	50	60	70	90	70
(3) Reading	40	50	60	80	60
Day 2					
(1) Reading	50	50	70	80	60
(2) Reading	40	40	60	80	60
(3) Reading	40	40	60	70	60
Day 3					
(1) Reading	50	50	70	80	60
(2) Reading	40	50	60	70	60
(3) Reading	40	40	60	70	50

**Table:** (b) The recording between Sessions 22 and 23

	Field 1	Field 2	Field 3	Field 4	Field 5
Day 1					
(1) Reading	50	50	70	80	70
(2) Reading	50	60	70	90	70
(3) Reading	40	50	70	70	60
Day 2					
(1) Reading	50	50	70	80	60
(2) Reading	40	50	60	70	60
(3) Reading	40	40	60	70	50
Day 3					
(1) Reading	40	50	60	70	60
(2) Reading	30	40	60	60	50
(3) Reading	30	40	50	60	40

**Table:** (c) The recording between Sessions 25 and 26 (the last session)

	Field 1	Field 2	Field 3	Field 4	Field 5
Day 1					
(1) Reading	30	30	50	60	50
(2) Reading	30	30	40	40	50
(3) Reading	20	30	40	40	40
Day 2					
(1) Reading	30	30	40	40	50
(2) Reading	30	30	40	30	40
(3) Reading	20	20	30	20	20
Day 3					
(1) Reading	20	30	20	30	20
(2) Reading	30	30	20	20	20
(3) Reading	20	30	20	20	20
